# Smoking, Symptoms Improvement, and Total Antioxidant Capacity in Patients with Drug-naive First-episode Schizophrenia: A Prospective Cohort Study

**DOI:** 10.2174/1570159X22666231019105328

**Published:** 2023-10-19

**Authors:** Zhiyong Gao, Meihong Xiu, Jiahong Liu, Fengchun Wu, Xiangyang Zhang

**Affiliations:** 1 The Affiliated Kangning Hospital of Wenzhou Medical University Zhejiang Provincial Clinical Research Center for Mental Disorder, Wenzhou, China;; 2 Peking University Huilongguan Clinical Medical School, Beijing Huilongguan Hospital, Beijing, China;; 3 Department of Psychiatry, The Affiliated Brain Hospital of Guangzhou Medical University, Guangzhou, China;; 4 Department of Biomedical Engineering, Guangzhou Medical University, Guangzhou, China;; 5 Guangdong Engineering Technology Research Center for Translational Medicine of Mental Disorders, Guangzhou, China

**Keywords:** Schizophrenia, smoking, risperidone, total antioxidant capacity, ANFE, PANSS

## Abstract

**Background:**

It has been hypothesized that smoking is associated with the severity of negative symptoms. Until now, no studies have investigated whether the impact of smoking on negative symptoms is dependent on antioxidants. This study was designed to evaluate the effect of smoking on therapeutic response and total antioxidants capacity (TAOC) in antipsychotic-naïve first-episode (ANFE) patients.

**Methods:**

The severity of the patient’s symptoms was assessed using the Positive and Negative Syndrome Scale (PANSS). A total of 237 ANFE patients were recruited and treated with risperidone (oral tablets, 4-6 mg/day twice a day) for 12 weeks. PANSS was assessed at baseline and a 12-week follow-up. Plasma TAOC levels were also assayed at baseline and week 12.

**Results:**

Relative to nonsmokers with ANFE SZ, smokers had higher PANSS negative subscores. There was no significant difference in TAOC changes after 12 weeks of treatment with risperidone between smokers and non-smokers. However, we found greater improvement in negative symptoms in smokers compared to non-smokers. Further analysis in smokers with SZ demonstrated that improvements in negative symptoms were not associated with changes in TAOC.

**Conclusion:**

Our study suggested that smoking affected the severity of baseline negative symptoms and further contributed to their reduction after risperidone treatment. However, improvement in negative symptoms was not dependent on the changes in TAOC.

## 1 INTRODUCTION

Schizophrenia (SZ) is a debilitating mental disorder characterized by various clinical symptoms [1-4]. The prevalence of smoking among patients with chronic SZ is estimated to be approximately about 14-88% in different countries, almost 2 times higher than the general population [5, 6]. Nicotine is one of the main psychoactive components of cigarettes, which regulates brain function through its interactive effect with the nicotinic acetylcholine receptors [7, 8]. However, the exact effect of smoking on SZ remains unclear.

Antipsychotics are the main treatment option to alleviate the clinical symptoms in patients affected by SZ [9]. Studies have shown that nicotine may be used as self-medication for clinical symptoms of SZ [10]. Nicotine may compensate for cognitive impairments of SZ by improving cognitive functioning, such as attention and visuospatial memory [11-13]. In addition, smoking can also reduce anxiety and depression symptoms because it alleviates unpleasant withdrawal symptoms [14]. A previous study speculated that anxiety reduction and sedation were the motivators for smoking in SZ [15]. However, a study by Goff *et al.* reported higher scores on the Brief Psychiatric Rating Scale (BPRS) and more hospitalizations among smokers [16].

Preliminary evidence suggests that excessive free radical production plays a critical role in the pathology of SZ [17, 18]. Free radicals are known to cause lipid, DNA, and protein damage in the body [19]. To combat and neutralize excess free radicals, complex antioxidants have been reported to be up-regulated in patients with SZ [20]. The body’s antioxidant defense system is dynamic and responsive to harmful oxidative stress and abnormal redox homeostasis. Antioxidants include enzymatic (catalase, glutathione peroxidase, and superoxide dismutase) and non-enzymatic (uric acid, vitamin C, glutathione, β-carotene, albumin) molecules to scavenge oxidative stress and prevent its deleterious effect once produced [21]. The imbalance between free radicals and antioxidants in favor of free radicals may contribute to oxidative stress in neurons [22]. An abnormal antioxidant defense system was found in the serum, plasma and brains of patients with SZ [4, 23, 24].

Cigarette smoke releases a complex mixture of 4000 to 7000 chemicals, including a high concentration of free radicals and other oxidants, which may increase lipid peroxidation and conversion of polyunsaturated fatty acids to hydroperoxides, endoperoxides, aldehydes and alkanes [25, 26]. Oxidative stress from smoking can be further enhanced in the lungs by the release of free radicals from macrophages [27, 28]. Cigarette smoke can cause oxidative stress not only by enhancing free radicals in smoke but also by weakening antioxidants [29]. Impaired antioxidant defense system in the blood of smokers, such as the activities of SOD, CAT, glutathione peroxidase, glutathione transferase and glutathione reductase, have been reported [30]. In addition, vitamin C levels were 40% lower in smokers than in non-smokers [31]. Other studies on smokers also revealed lower levels of uric acid in smokers than in healthy controls [32-34]. Specifically, a recent study reported that cigarette smokers had a lower average total antioxidant capacity (TAOC) than nonsmokers [35].

TAOC is a valid indicator of total antioxidant concentration in the body [36]. Studies have found abnormal TAOC in patients with SZ, which was associated with the severity of symptoms [23, 24, 37-39]. Antipsychotics have been reported to alleviate psychotic symptoms in part by modulating antioxidant defense systems [40]. There is indeed a growing body of evidence that antipsychotics can regulate TAOC [41]. Risperidone is an atypical antipsychotic for the treatment of patients with SZ [42], which is usually administered as oral tablets (4-6 mg/day) twice a day. We chose risperidone as an antipsychotic because it does not exhibit significant α7 nicotinic acetylcholine receptor agonism or 5-HT_3_ receptor antagonism [43]. Also, smoking has been reported to have a possible effect on the pharmacokinetics and metabolism of antipsychotics [44]. Cigarette smoking induces CYP1A2 activity, leading to a decrease in risperidone plasma levels [45]. However, to date, no studies have investigated the impact of smoking on TAOC and the pathophysiology of SZ after treatment with risperidone. We hypothesize that smoking affects the therapeutic response to risperidone, which was dependent on TAOC in patients with ANFE SZ. Therefore, we aimed to explore (1) whether TAOC was different between smokers and nonsmokers; (2) whether smoking had an impact on clinical symptoms after risperidone monotherapy for 12 weeks; and (3) whether improvement in symptoms was dependent on the changes in TAOC.

## 2 MATERIALS AND METHODS

### 2.1 Participants

Two hundred and thirty-seven ANFE patients with SZ were recruited from Beijing hui-long-guan Hospital and Henan Zhu-Ma-Dian Hospital. All patients met the DSM-IV diagnosis of SZ based on the Structured Clinical Interview I for DSM-IV (SCID-I) criteria. Patients with any other major Axis I disorder were excluded from this study. Patients were first-episode and drug-naïve, were between 16 and 45 years of age, and had a cumulative use of antipsychotic drugs < 14 days. The duration of the illness was no more than 5 years. One hundred and twenty-five unrelated, age- and sex-matched healthy controls (HC) participated in the present study. We also excluded HCs with psychiatric disorders through the SCID interviews.

In addition, we obtained a complete set of medical history, physical examinations, and laboratory tests from patients and HCs. Participants were excluded if they had major medical comorbidities (*i.e*., infection, diabetes and hypertension), cerebrovascular diseases, took over-the-counter antioxidants or were pregnant. HC subjects were also assessed for personal history of psychiatric disorders based on the SCID. If HCs had a first-degree relative diagnosed with a mental disorder, they were excluded.

The present study was approved by the Ethical Committee of Beijing Hui-long-guan Hospital in accordance with the Declaration of Helsinki. All subjects provided written informed consent for participation in this study.

ANFE patients with SZ were treated with oral risperidone (4-6 mg/day) for 12 weeks. Risperidone was administered as oral tablets twice a day. The risperidone treatment started with 1 mg/day, with increments of 1 mg every 3 days according to the reaction and tolerance of patients. Finally, the dose of risperidone usually reached 4-6 mg/day over 2 weeks.

### 2.2 Assessments and Outcomes

Six psychiatrists assessed the clinical symptoms using the Positive and Negative Syndrome Scale (PANSS). After a training course on how to use this scale, an inter-observer correlation coefficient greater than 0.8 for PANSS total score was maintained across repeated evaluations. Clinical symptoms were assessed at baseline and the 12-week endpoint.

A smoker is defined as someone who smokes at least one cigarette a day and has been smoking for more than one year. Nonsmokers are defined as people who have never smoked or have smoked less than 100 cigarettes in their lifetime [46].

### 2.3 Determination of TAOC Activity

Blood samples were obtained from all participants after fasting for 12 hours and stored at -80°C until TAOC was assayed. TAOC was determined by two experienced technicians through the kits (Nanjing Jiancheng Bioengineering Institute, Nanjing, China), who were blinded to the participant’s status [47]. In brief, TAOC was measured by the FRAP method, expressed as reductants from Fe^3+^ to Fe^2+^, which were chelated by 2,4,6-tri-pyridyl-s-triazine (TPTZ) to form a Fe^2+^-TPTZ complex. The complex was absorbed in 593 nm and detected by a Multiskan microplate reader.

### 2.4 Statistical Analysis

Demographic characteristics between smokers and nonsmokers with SZ were compared by *X*^2^ test and independent sample t-test. Analysis of covariance (ANCOVA) was performed to examine whether there was a significant difference in TAOC between HCs and patients at baseline. The covariables included sex, age and onset age, which have previously been shown to be associated with oxidative stress. We used the G*Power 3.1.9.2 program to run a power calculation and determine the sample size.

Analyses of the main outcomes were conducted by intention-to-treat analysis (ITT), including all patients who provided baseline and second-month assessments. Clinical symptoms and TAOC were compared between baseline and follow-up using paired t-tests. To investigate the impact of smoking status on the main outcomes and TAOC, a repeated-measures ANCOVA (Rm ANCOVA) was conducted with smoking status as a dependent variable adjusting for the age, sex and onset age. Pearson’s correlations or Spearman’s correlations were used to examine the correlation between the changes in TAOC and PANSS or between baseline TAOC and changes in PANSS in smokers and non-smokers, respectively. Changes in PANSS and biomarkers between follow-up and baseline were calculated by subtracting the baseline values from the follow-up values. Further linear regression was performed to determine whether the changes in TAOC were associated with improvements in clinical symptoms after adjusting for covariates in smokers and nonsmokers, respectively.

SPSS (Statistical Package for the Social Sciences) software was performed in all statistical analyses. Statistical significance was taken as *p* < 0.05.

## 3 RESULTS

### 3.1 Demographic and Clinical Data at Baseline between Smokers and Non-smokers with SZ

There were 67 smokers and 170 non-smokers in the patient group and 45 smokers and 80 non-smokers in the HC group. The demographic and clinical characteristics between smokers and non-smokers in patients are shown in Table **1**. There were significant differences in age, age of onset, and negative symptoms between smokers and non-smokers with SZ (all *p* < 0.05) (Table **1**). Among patients, smokers were older, had a higher age of onset and had more severe negative symptoms than nonsmokers.

As reported in our previous study, baseline TAOC was significantly higher in ANFE patients than HCs [48]. Baseline TAOC was not associated with age, onset age, and years of education (all *p >* 0.05). There was no significant difference in TAOC between smokers and non-smokers with SZ (216.5 ± 68.3 *vs.* 225.6 ± 68.4, F = 0.9, *p =* 0.36). Moreover, we found no difference in TAOC between smokers and non-smokers in the whole subjects (*p >* 0.05).

### 3.2 Symptoms Improvements and TAOC after Treatment between two Groups

**Fig. (1):**
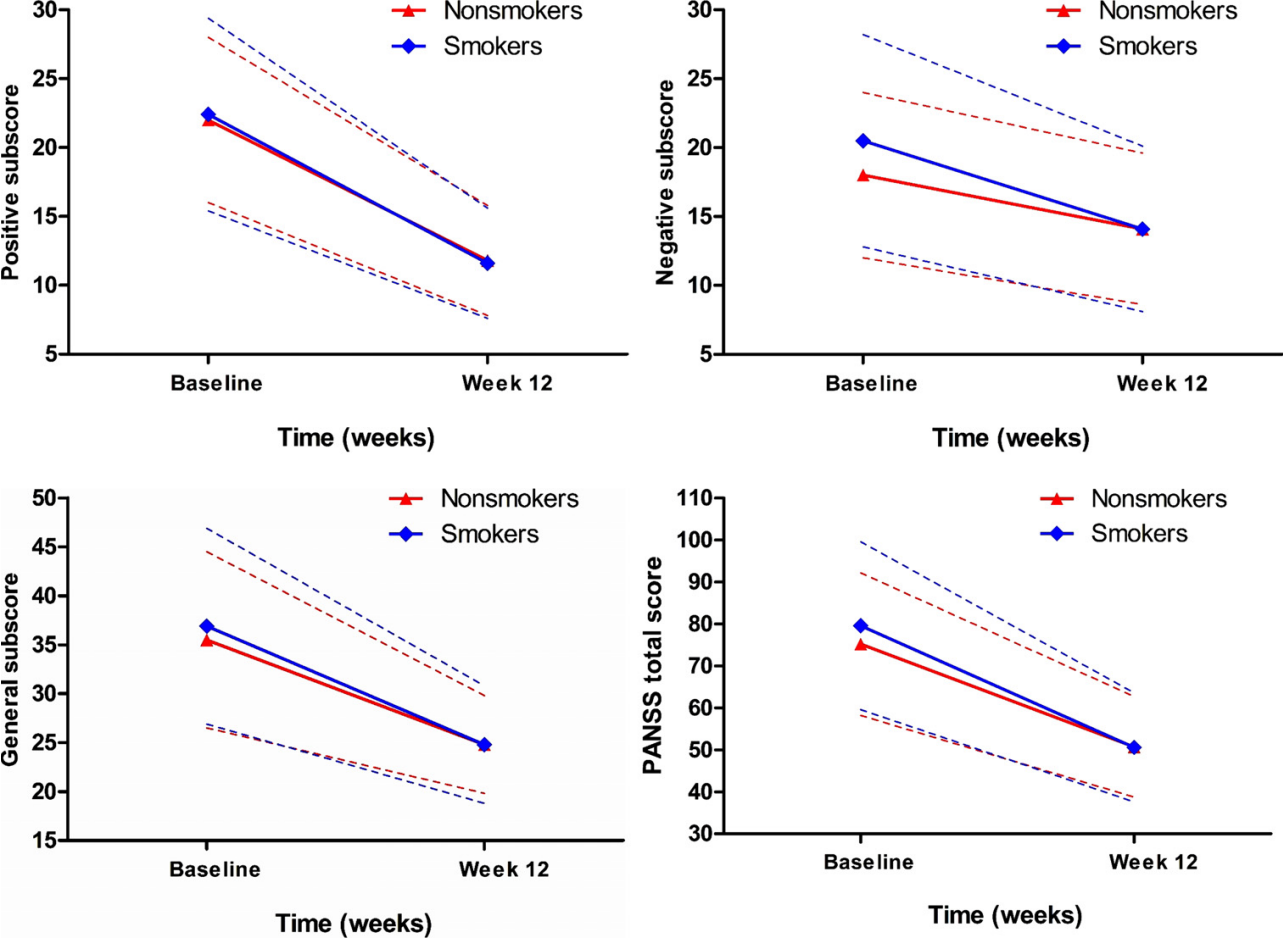
There were significant interaction effects of time and smoke on negative symptoms in patients with schizophrenia after treatment with risperidone (F = 11.9, *p* = 0.001) and PANSS total score (F = 4.4, *p* = 0.036).

**Fig. (2):**
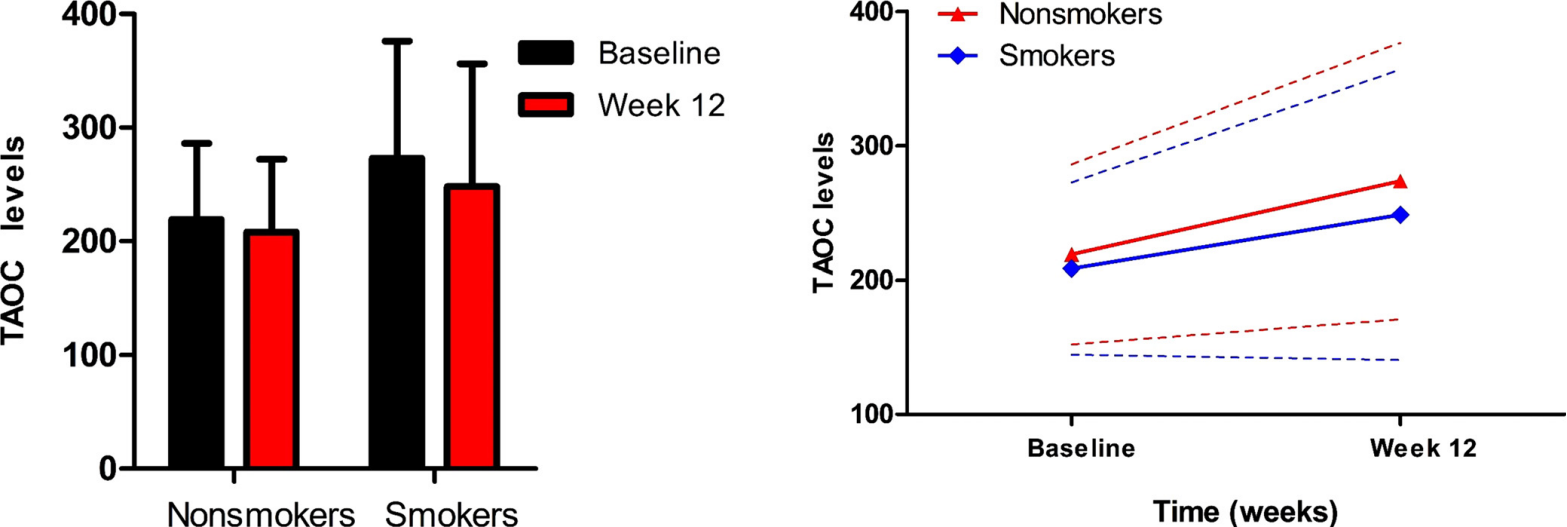
There was no significant interaction effects of time and smoke on TAOC levels in patients with schizophrenia after treatment with risperidone (*p* > 0.05).

Rm ANCOVA analysis found that after treatment for 3 months, PANSS total score and all its subscores were significantly decreased after controlling for age, sex, and onset age (*p <* 0.05). There were significant interaction effects of time and smoke on negative symptoms (F = 11.9, *p =* 0.001) and PANSS total score (F = 4.4, *p =* 0.036) (Table **2** and Fig. **1**), after controlling for age, sex and onset age. In smokers, negative symptoms and PANSS total score were significantly reduced after treatment (N: 20.5 ± 7.7 *vs.* 14.1 ± 6.1, changes: 6.4 ± 7.6, t = 6.5, *p <*0.001; Total: 79.6 ± 20.2 *vs*. 50.6 ± 13.4, changes: 29.1 ± 20.2, t = 11.0, *p <*0.001). Moreover, significant improvements in negative symptoms and total score were found in non-smokers (N: 18.0 ± 6.6 *vs*. 14.1 ± 5.5, changes: 4.0 ± 5.5, t = 8.6, *p <*0.001; Total: 75.2 ± 17.1 *vs*. 50.7 ± 12.7, changes: 24.4 ± 17.6, t = 16.7, *p <*0.001) (Fig. **1**). Further analysis found that smokers showed greater improvement in negative symptoms and PANSS total score than non-smokers after controlling for covariates (all *p <* 0.05). However, there was no significant interaction effect of time and smoke and the main effect of smoke on positive symptoms, general psychopathology or TAOC (all *p >* 0.05) (Table **2** and Fig. **2**).

### 3.3 Association between Smoking, Baseline TAOC, Changes in TAOC and Improvements in Clinical Symptoms

Among smokers and non-smokers with SZ, baseline TAOC was negatively correlated with the improvements in positive symptoms, general psychopathology, and total PANSS score (all *p <* 0.05) (Table **3**). In addition, regression analysis supported the association of baseline TAOC with the improvements in these clinical symptoms among both groups after controlling for age, sex and onset age (Table **3**). Smoke did not affect the association between baseline TAOC and symptom improvement in patients with SCZ.

We further analyzed whether changes in TAOC were associated with improvements in clinical symptoms among smokers and non-smokers with SZ. The results showed that smoking showed no impact on the association between the increase in TAOC and the improvement in PANSS total score or its subscores (all *p >* 0.05).

## 4 DISCUSSION

This is the first and largest study to evaluate the impact of smoking on TAOC and symptoms in patients with SZ after 12 weeks of risperidone monotherapy. This study had several findings, which are as follows:

1. There was no difference in plasma TAOC between smokers and non-smokers with SZ.
2. After 12 weeks of treatment, TAOC increased significantly in both smokers and non-smokers, and baseline TAOC was associated with a reduction in clinical symptoms after risperidone treatment.
3. Smokers showed greater improvement in negative symptoms and total score than non-smokers.

Cigarette smoke is highly prevalent in SZ, but its effect on symptoms and relationship to oxidative stress in SZ is not known [49]. Our finding of no significant difference in baseline TAOC between smokers and non-smokers suggests that smoking had no impact on plasma TAOC concentrations in the early stage of SZ. Our study was consistent with previous studies on SZ [50, 51]. For example, Ballesteros *et al.* found no significant effect of smoking and medications on the concentrations of glutathione (GSH) [51]. However, other studies have reported conflicting findings. Gould *et al.* found that tobacco smoking causes an adaptive antioxidant response and increases systemic glutathione production [52]. Zhang *et al.* reported an increase in the antioxidant enzymes in patients with SZ who smoked [53]. The inconsistency may be due to the differences in antioxidant molecules, sample type, and disease progression.

In addition, we failed to find a significant difference in the increase of TAOC between smokers and non-smokers after treatment with risperidone for 3 months. The increase in TAOC indicated that antipsychotics might upregulate antioxidants early in treatment, independent of smoking. To the best of our knowledge, this is the first study to directly assess the association between smoking and changes in TAOC following antipsychotic treatment in ANFE patients with SZ. The finding of increased TAOC suggested that the elaborate redox system in favor of antioxidants exists in the early stage of treatment in SZ. As a compensatory mechanism, the elevated TAOC was caused by excess free radicals. However, this elevation was not influenced by the smoking status of patients. Therefore, future studies are warranted to evaluate the effect of antipsychotic drugs on TAOC and other antioxidant molecules in SZ.

We further found that smoking affected the improvement of negative symptoms during the treatment with risperidone in patients and that smokers showed a greater improvement in negative symptoms than non-smokers. Our findings were in line with previous clinical and animal studies [54, 55]. Smoking has indeed been reported to be used as self-medication because of its direct pharmacotherapeutic effects, such as sedative, stimulant and sensorimotor manipulation properties [56]. Increased activity of the midbrain dopaminergic and reward systems has been reported after systemic administration of nicotine in rats [54], which may be a potential mechanism for reducing negative symptoms in SZ. Smoking helps to confront problems, gives pleasure, and alleviates the symptoms [57]. Nicotine plays a critical role in psychiatric disorders, probably because it increases the activity of the liver cytochrome P450 enzyme and increases the metabolisms of antipsychotic drugs. In addition, smoking reduces dopamine degradation, enhances dopaminergic activity, and activates reward-related neural regions in the frontal-subcortical circuitry (mesocortical dopamine pathways). Thus, smoking increases dopamine levels by causing dopamine release and inhibiting its degradation, which then further reduces the negative symptoms in patients with SZ [58-60]. Therefore, in smokers, risperidone is more effective than in non-smokers for negative symptoms. Although smoking did not alter TAOC, it is unclear whether smoking alters other antioxidant molecules. In addition, we measured TAOC only in the blood rather than in CNS or CSF. Therefore, this study is preliminary, and the association of TAOC with smoking and clinical symptoms in SZ merits further investigation.

Notably, smokers with SZ showed greater negative symptoms than non-smokers at baseline. We do not know the exact reason for this finding. However, it has been reported that chronic nicotine exposure desensitizes nicotinic receptors and reduces the cholinergic activity in the prefrontal cortex of patients with SZ, which may adversely affect the negative symptoms [61]. Indeed, cigarette smoke is a complex process involving psychopathological, biochemical and neuropharmacological domains in patients with SZ [62]. Previous findings in the literature were inconsistent. There were few clinical data suggesting that nicotine helps to reduce negative symptoms in patients suffering from SZ [63]. Dixon *et al.* (2007) reported a decrease in negative symptoms and an increase in positive symptoms in patients affected by SZ with higher severity of smoking [64]. On the contrary, Barnes *et al.* showed no significant difference in clinical symptoms between smoking and non-smoking patients [65]. In particular, Krishnadas *et al.* found that patients with severe nicotine dependence had higher scores on the positive PANSS subscale, while patients with mild to moderate dependence had higher scores on the negative subscale [66]. Therefore, further studies on the role of smoking and nicotine in the clinical symptoms of SZ are warranted.

The strength of our study included the relatively large sample size, a prospective design, and the use of drug naïve first-episode patients with SZ. However, several limitations should also be noted in this study. First, only TAOC levels were measured in this study. Many enzymatic and non-enzymatic antioxidants were not determined, which need to be measured in future studies. Second, some other risk factors should also be considered in future research, such as dietary style, lifestyle, and physical exercise. Third, the number of cigarettes smoked and the severity of nicotine dependence were missed; therefore, the associations between the severity of nicotine and improvements in clinical symptoms and TAOC levels were not investigated. Fourth, another limitation was a lack of data on BMI and insulin resistance, which have been reported to have a significant role in response to treatment, cognitive dysfunction, and complex inflammatory and oxidant processes in first-episode patients with SZ. Fifth, only the effect of risperidone was examined in this study. Future studies should investigate whether other antipsychotics have similar findings.

## CONCLUSION

In summary, this study found a significant effect of smoking on the improvement of negative symptoms after treatment with risperidone in patients with ANFE SZ. However, smoking showed no impact on TAOC or the changes after treatment. Therefore, in clinical practice, there is no need to pay particular attention to smoking cessation in patients with SZ, considering that smoking shows a non-significant effect on total antioxidant status but further improves negative symptoms. However, it is only a preliminary study and needs replication in other conventional antipsychotics. Future studies are also warranted to investigate the additive effect of smoking on the clinical symptoms and redox system in the early treatment of SZ.

## Figures and Tables

**Table 1:** Demographic characteristics and clinical data in antipsychotics-naive first episode (ANFE) patients with schizophrenia (SZ) and healthy controls.

**Variable**	**SZ**		*** p*-value**	**HC**		*** p*-value**
	**Non-smokers ** **(n = 170)**	**Smokers ** **(n = 67)**		**Non-smokers ** **(n = 80)**	**Smokers ** **(n = 45)**	
Sex (male/female)	70/100	58/9	< 0.001	37/43	40/5	< 0.001
Age (years)	26.7 ± 9.2	30.6 ± 8.6	0.03	26.5 ± 7.4	29.6 ± 8.6	0.03
Education (years)	9.3 ± 3.9	8.2 ± 3.8	0.05	10.9 ± 3.0	9.3 ± 3.0	0.007
Age of onset (years)	25.4 ± 9.1	28.6 ± 9.0	0.016	-	-	-
PANSS score		-		-	-	-
P N G Total score	21.6 ± 6.1 18.2 ± 6.5 35.0 ± 9.3 74.6 ± 16.2	22.3 ± 6.9 20.6 ± 7.6 36.8 ± 10.1 79.5 ± 19.5	0.46 0.016 0.19 0.05	-	-	-

**Note:** *PANSS* the positive and negative syndrome scale; *P* positive symptoms; *N* negative symptoms; *G* general psychopathology.

**Table 2:** Comparisons of clinical symptoms and TAOC levels before and 3 months after risperidone monotherapy using Rm ANCOVA.

**-**	**Baseline**		**12-week follow-up**		**Effect^a^**		
	**Non-smokers ** **n = 142^b^**	**Smokers ** **n = 59^b^**	**Non-smokers ** **n = 142^b^**	**Smokers ** **n = 59^b^**	**Time ** **F(p)^a^**	**Smoke ** **F(p)^a^**	**Interaction ** **F(p)^a^**
P	22.0 ± 6.3	22.4 ± 7.1	11.8 ± 4.4	11.6 ± 4.3	10.4(0.001)	0.2(0.70)	1.2(0.28)
N	18.0 ± 6.6	20.5 ± 7.7	14.1 ± 5.5	14.1 ± 6.1	5.8(0.017)	1.6(0.27)	11.9(0.001)
G	35.5 ± 9.8	36.9 ± 10.5	24.8 ± 5.7	24.8 ± 6.7	6.2(0.014)	0.1(0.71)	1.5(0.22)
Total score	75.2 ± 17.1	79.6 ± 20.0	50.7 ± 12.7	50.6 ± 13.4	10.7(0.001)	0.7(0.40)	4.4(0.036)
TAOC	219.0 ± 67.3	208.4 ± 64.7	273.6 ± 103.5	248.4 ± 108.3	41.5(< 0.001)	1.0(0.32)	2.5(0.12)

**Note:** ^a^The *p* values were adjusted for sex, age, and onset age, ^b^These numbers were the sample size after dropout.

*P* positive symptoms; *N* negative symptoms; *G* general psychopathology; *TAOC* total antioxidants capacity.

**Table 3:** Correlation and regression analysis of the associations between baseline TAOC levels.

	**Pearson Correlation Analysis**		**Regression Analysis**		
**Smokers**					
	**r**	***p***	**β**	**t**	***p***
P	-0.44	**0.001**	-0.39	-3.13	**0.003**
N	-0.15	0.25	-0.12	-0.94	0.35
G	-0.36	**0.006**	-0.31	-2.35	**0.02**
Total score	-0.39	**0.002**	-0.34	-2.67	**0.01**
**Non-smokers**					
	**r**	***p***	**β**	**t**	***p***
P	-0.27	**0.001**	-0.28	-3.30	**0.001**
N	-0.05	0.57	-0.23	-0.27	0.79
G	-0.17	**0.04**	-0.16	-1.89	0.06
Total score	-0.20	**0.014**	-0.19	-2.25	**0.026**

**Note:** *P* positive symptoms; *N* negative symptoms; *G* general psychopathology.

## Data Availability

The datasets generated during and/or analyzed during this study are available from the corresponding author upon reasonable request.

## LIST OF ABBREVIATIONS

ANFE: Antipsychotic-naïve First-episode
BPRS: Brief Psychiatric Rating Scale
HC: Healthy Controls
PANSS: Positive and Negative Syndrome Scale
SZ: Schizophrenia
TAOC: Total Antioxidants Capacity

## STANDARDS OF REPORTING

STROBE guidelines and methodology were followed.
